# The relationship between motor competence and health-related fitness in children and adolescents

**DOI:** 10.1371/journal.pone.0179993

**Published:** 2017-06-28

**Authors:** Carlos Luz, Luís P. Rodrigues, An De Meester, Rita Cordovil

**Affiliations:** 1 Escola Superior de Educação de Lisboa, Instituto Politécnico de Lisboa, Lisboa, Portugal; 2 Faculdade de Motricidade Humana, Universidade de Lisboa, Lisboa, Portugal; 3 Research Center in Sports, Health and Human Development (CIDESD), Vila Real, Portugal; 4 Escola Superior de Desporto e Lazer de Melgaço, Instituto Politécnico de Viana do Castelo, Melgaço, Portugal; 5 Department of Movement and Sports Sciences, Ghent University, Ghent, Belgium; 6 CIPER, Faculdade de Motricidade Humana, Universidade de Lisboa, Lisboa, Portugal; Kent State University, UNITED STATES

## Abstract

**Background and aims:**

In the last twenty years, there has been increasing evidence that Motor Competence (MC) is vital for developing an active and healthy lifestyle. This study analyses the associations between motor competence and its components, with health-related fitness (HRF).

**Methods:**

A random sample of 546 children (278 males, mean = 10.77 years) divided into four age groups (7–8; 9–10; 11–12; 13–14 years old) was evaluated. A quantitative MC instrument (evaluating stability, locomotor and manipulative skills), a maximal multistage 20-m shuttle-run test and the handgrip test, height and BMI were used in the analyses. Pearson correlations and standard regression modelling were performed to explore the associations between variables.

**Results:**

Moderate to strong significant correlations (0.49 < r < 0.73) were found between MC and HRF, for both sexes, and correlation values were stable across the age groups. The MC model explained 74% of the HRF variance, with the locomotor component being the highest predictor for the entire sample (*β* = .302; *p* < .001). Gender-related differences were found when boys and girls were analysed at each age group. Locomotor MC for girls was the most consistent significant predictor of HRF across all age groups (0.47 < *β* < 0.65; all *p*≤.001). For boys, significant predictors were locomotor and manipulative MC (0.21 < *β* < 0.49; all *p* < .05) in the two younger age groups (7–8 and 9–10 years) and stability (0.50 < *β* < 0.54; all *p*≤.001) for the older two age groups (11–12 and 13–14 years).

**Conclusion:**

These results support the idea that: (1) the relationship between overall MC and HRF is strong and stable across childhood and early adolescence; (2) when accounting for the different MC components, boys and girls show different relationship patterns with HFR across age.

## Introduction

Research has demonstrated a trend in recent years of children spending more time engaging in sedentary behaviours [1,2], and spending less time being physically active [3]. Furthermore, there has been a decline in children’s motor competence (MC) and health related fitness (HRF) [4–6] and at the same time an alarmingly high prevalence of overweight and obesity in children [7].

Motor competence relates to the development and performance of human movement [8]. In the literature, MC has encompassed a wide variety of terms such as fundamental motor skill or movement, motor proficiency or performance, motor ability and motor coordination [9]. In this study, and as proposed by the theoretical framework developed by Gallahue and colleagues [10], MC will be defined as a person’s ability to be proficient in a broad range of locomotor, stability and manipulative skills [11,12]. Recent research emphasizes the importance of MC for developing an active and healthy lifestyle [8,9,13,14]. Several cross-sectional and longitudinal studies in children and adolescents found positive associations among MC and physical activity [14,15], among MC and HRF [16–18], and an inverse association between MC with weight status [5,19,20] and sedentary behavior [2,21]. Additionally, MC has been found to be an important predictor of physical activity in childhood [22] and adolescence [23].

The theoretical model developed by Stodden and colleagues [8] proposes a reciprocal and developmentally dynamic relationship between MC and HRF with the strength of this relationship increasing over time. A recent study found that the correlation values between manipulative skills (i.e. gross motor skills that use hands or feet to move or manipulate an object) and HRF increases over time, suggesting that these skills may play a role in the association between MC and HRF [17]. A recent review by Robinson and colleagues’ [9] addressing the Stodden et al. [8] model hypotheses also supports the association between MC and HRF. However, conclusive evidence that these associations become stronger with increasing age has not been fully addressed.

The purpose of this study was to analyse the relationships between MC components and HRF in a large sample of children from 7 to 14 years. It is hypothesized that MC and its components will be positively associated with HRF, with greater strength in older age groups [8,9]. Additionally, manipulative skills are expected to have a decisive role in the associations between MC and HRF [17,24]. To our knowledge, this study is the first to examine these variables using a quantitative instrument [12] that provides an overall MC composite value derived from locomotor, stability and manipulative components [10].

## Methods

### Participants

A random sample of 546 children (278 boys, 50.9%) with a mean age of 10.8 years (SD = 2.3; range 7–14 years) participated in this study. The sample was divided into 4 age groups by 2-year intervals (see Table 1). Children were selected from public schools in different municipalities in the Lisbon district and had no motor, cognitive or health impairments that could affect their performance on the motor or fitness tests. Two trained physical education teachers collected the data during regularly scheduled classes from april to june 2015. The ethical committee of the Faculdade de Motricidade Humana ensured and approved the conformity procedures regarding scientific research involving human beings. Written informed consent was obtained from schools and the parents of all participants.

**Table 1:** Mean and standard deviation of boys and girls by age group for MC, HRF, height and BMI.

	Boys				Girls			
	7–8 years n = 81	9–10 years n = 72	11–12 years n = 66	13–14 years n = 59	7–8 years n = 79	9–10 years n = 71	11–12 years n = 54	13–14 years n = 64
Var.	Mean±SD	Mean±SD	Mean±SD	Mean±SD	Mean±SD	Mean±SD	Mean±SD	Mean±SD
Stability^1^ (t-score)	43.5±7.0	48.4±7.9	53.1±7.5	60.8±8.8	41.1±7.3	49.5±8.5	51.4±6.6	57.2±9.5
Locomotor^1^ (t-score)	46.2±6.6	50.2±7.6	54.9±9.2	62.9±8.8	40.5±8.1	47.3±7.2	50.3±8.1	52.0±8.1
Manipulative^1^ (t-score)	46.0±5.3	52.4±5.8	58.4±6.9	66.5±8.7	38.1±4.9	44.4±4.5	48.2±4.2	51.0±5.8
MC^2^ (t-score)	44.6±5.8	50.3±6.3	56.2±7.7	65.1±8.4	38.6±6.5	46.8±6.0	49.9±5.6	53.8±7.1
HRF^3^ (t-score)	45.3±4.9	49.1±5.1	53.6/±7.2	62.9±8.5	42.2±3.6	46.7±4.2	50.4±5.0	54.5±4.6
Height (cm)	127,9±6.2	138.7±6.4	151.9±9.4	162.5±9.0	127.6±6.7	139.4±7.5	153.4±8.8	159.8±6.9
BMI (kgm^-1^)	17.1±1.9	19.0±3.4	20.1±3.5	20.5±3.9	17.5±2.5	18.7±3.3	20.2±4.7	22.1±4.4

1—Stability, locomotor and manipulative category scores were calculated as the sum of the t-scores of the two tasks.

2—Total MC was calculated as the mean of the t-scores for all components.

3 –An HRF was calculated as the mean of the t-scores of the PACER test and the handgrip test

### Measures

#### Health-related fitness

The PACER and the handgrip tests were used to evaluate cardiorespiratory fitness and upper body strength, respectively. The PACER test involved a progressive shuttle run (20 meters) test protocol with an increasing cadence [25]. To help children establish the rhythm of the run, one experienced PE teacher, that was able to maintain the appropriate running speed for each test phase, ran alongside the children providing encouragement.

The handgrip test is a commonly used test for assessing muscular strength [26]. Each participant started from a standing position and using the dominant hand, squeezed the dynamometer with maximum isometric effort maintained for about 5 seconds. The best result after 3 attempts was recorded as the final score. An HRF index was calculated from the mean of the t-scores of the PACER test and the handgrip test.

#### Motor competence

MC was evaluated with a valid quantitative instrument developed by Luz and colleagues [12]. This instrument is composed of two tests for each MC category (stability, locomotor, and manipulative). Stability tests: **Shifting platforms** required subjects to move sideways for 20s using two wooden platforms (25cm x 25cm x 2cm with four 3.7 cm feet at the corners). Each successful transfer from one platform to the other is scored with two points (one point for moving the platform; one point for moving into the platform). Participants completed two trials and the best score was recorded. **Lateral Jumps** required subjects to jump sideways with two feet together over a small wooden beam (60 cm length×4 cm high ×2 cm width) located in the middle of a rectangular surface (100 cm length x 60 cm width) as fast as possible for 15s. Each correct jump (two feet together, without touching outside the rectangle, and without stepping in the wooden beam) was scored 1 point and the best score was recorded. Locomotor tests: The **shuttle run (SHR)** required subjects to run at maximal speed between the starting line and a line placed 10 meters away. Beginning at the starting line, subjects ran to the opposite line, picked up a block of wood, ran back and placed the block beyond the starting line. Subjects then ran back to retrieve the second block and carry it back across the starting line to finish the test. The best time of the two trials was recorded. The **standing long jump (SLJ)** required subjects to jump with both feet simultaneously as far as possible. The best score of 3 attempts was the longest distance in cm between the starting line and the back of the heel at landing. Manipulative tests: **Throwing velocity** required subjects to throw a ball against a wall at maximum speed using an overarm action with a preparatory balance (one or two steps). For children between 7 and 10 years old, a tennis ball was used (diameter: 6.5 cm; weight: 57g). For children 11 years old and older, a baseball was used (diameter: 7.3 cm; weight: 142g). Peak velocity was measured in m/s with a velocity radar gun (e.g. Pro II Stalker radar gun). Every participant performed three trials, with the final score being the best result. Kicking Velocity required subjects to kick a soccer ball against at wall at maximum speed using a preparatory balance (one or two steps). For children 7- and 8-years-old, a soccer ball n°3 was used (circumference: 62cm, weight: 350g). For children 9- and 10-years-old, a soccer ball n°4 was used (circumference 64cm, weight: 360g). For subjects older than 10 years-of-age, a soccer ball n°5 (circumference 68cm, weight: 410g) was used. Ball peak velocity was measured in m/s with a velocity radar gun (e.g. Pro II Stalker radar gun). Every subject performed three trials, with the final score being the best result.

Stability, locomotor and manipulative category scores were calculated as the sum of the t-scores of the two tasks. SHR inverse t-values were used given that higher values (s) represented lower performance. Total MC was calculated as the mean of the t-scores for all categories.

#### Somatic variables

Subjects were asked to be barefoot and wear minimal sport clothes to measure their height and weight. Height was measured using a SECA stadiometer and weight was measured on a Tanita digital balance scale (BF-350 Total Body Composition Analyzer) according to a standardized anthropometric measurement protocol [27]. BMI was calculated using height and weight [(height (kg)/height (m2)].

### Procedures

Two testing sessions on separate days were needed to complete the somatic measures and the HRF and MC tests. On the first day, groups of five children completed the MC protocol in the following order: stability, locomotor, and manipulative tasks. On the second day, groups of 20 children completed the height and weight measures (somatic variables) and the HRPF tests (handgrip test and PACER). For all MC and HRPF tests, the PE teacher was expected to follow each respective test protocol. Participants were allowed to try each test once before the actual test administration. Motivational feedback was provided by the PE teachers, but verbal feedback on skill performance was not provided. The same procedures were used for all tests.

### Statistical analyses

Power analyses calculations for the prediction of HRF using five independent variables with an alpha set at p < .05, a predicted effect size of .30, and a power estimate of .80, yielded a sample size of 49, showing that the sample size for each age group and sex had enough statistical power to detect significant and meaningful effects.

Descriptive statistics (means and standard deviations) were calculated to characterize height, BMI, MC, and HRF by age group and sex. Pearson’s bivariate correlations were used to assess the relationships between the MC and HRF variables. To test if the relationship between MC and HRF differed across age groups, a one-way analysis of covariance (ANCOVA) was conducted with HRF as the dependent variable, age group as the independent variable, and age-by-manipulative, age-by-stability, and age-by-locomotor as the covariates. A hierarchical multiple regression analysis was performed on all participant data to measure the predicted variance of MC (independent variable) for HRF (dependent variable). In order to control for developmental status, gender-related differences, and somatic influence, age (decimal age), height, sex, and BMI were initially included in the regression analyses as independent variables, but collinearity problems between age and height resulted in the decision to keep age when analyzing the entire sample and height when analysing age groups. In the multiple regressions, and in order to accommodate for the presence of several predictors, adjusted R^2^ values were used to assess the amount of HRF variance explained by the independent variables. Separate standard regressions analyses for each age group’s HRF were analyzed using the individual MC components, height, and BMI as predictors. The testing for the normality of variables was assured before each regression, and their resulting residuals were analyzed in order to assure the assumption of normality. A maximal value of 0.2 was set for collinearity tolerance of the independent variables in the multiple regressions. All statistical analyses were conducted in SPSS version 20 and at a 0.05 level of significance.

## Results

Descriptive data indicated that all variables of interest increased across age groups in both sexes (see Table 1). Correlations among MC and HRF variables are presented in Table 2. In general, moderate to high correlations between MC and HRF (.49 to .73) were found for both sexes in all age groups. Of the three MC components measured, locomotor skills were found to have the strongest relationship with HRF across all age-groups and sexes; and manipulative skills were more often correlated with HRF in boys than girls (*p* < .05 for 1–8, 11–12, and 13–14 years-old). ANCOVA analyses revealed significant effects for all age-related covariates, namely age-by-stability (*F*(1, 529) = 24.90, *p* < .001), age-by-locomotor (*F*(1, 529) = 69.45, *p* < .001), and age-by-manipulative (*F*(1, 529) = 69.62, *p* < .001). Therefore, multiple regression analyses were performed for the entire sample and for age groups by MC component to further analyse the relationship between HRF and MC components.

**Table 2:** Pearson correlations between MC (components and total) and HRF by sex and age group.

**Boys**				
	**HRF**			
**Age (years)**	**7–8**	**9–10**	**11–12**	**13–14**
**Stability**	.48***	.40***	.68***	.70***
**Locomotor**	.60***	.62***	.64***	.51***
**Manipulative**	.67***	.49***	.50***	.59***
**MC**	.71***	.64***	.71***	.71***
**Girls**				
	**HRF**			
**Age (years)**	**7–8**	**9–10**	**11–12**	**13–14**
**Stability**	.60***	.47***	.28*	.61***
**Locomotor**	.97***	.60***	.57***	.56***
**Manipulative**	.54***	.37***	.20	.43***
**MC**	.73***	.62***	.49***	.68***

Note:

*p < .05

***p < .001

The hierarchical regression analysis for the entire sample is displayed in Table 3. In the first model, sex, age, and BMI were found to be significant predictors of MC, explaining 58% of the HRF variance. When including the MC composite in the second model and the MC components in the subsequent model, the explained variance increased to 74%. Age was kept as a significant predictor in all models and when tested (model 3), all the three MC components (stability, locomotor, and manipulative) were found to be significant predictors of the HRF behaviour.

**Table 3:** Multiple regressions for age, sex, BMI, MC, and MC components to HRF for the entire sample.

Step	Predictors	β	p	F	R^2^	p
1				243.5	.58	.000
	Age	.772	.000			
	Sex	-.262	.000			
	BMI	-.149	.000			
2				383.1	.74	.000
	Age	.264	.000			
	Sex	-.044	.078			
	BMI	.017	.517			
	MC	.649	.000			
3				257.2	.74	.000
	Age	.265	.000			
	Sex	-.026	.399			
	BMI	.018	.522			
	Stability	.178	.000			
	Locomotor	.302	.000			
	Manipulative	.257	.000			

Standard regressions were also performed for each age group and by sex with the MC components, height, and BMI as independent variables (see Table 4). Including height and BMI as independent variables in each regression allowed us to control for their effects on HRF, which provided a cleaner image of the real effects of the independent variables of interest (MC components). Separate analyses were run for boys and girls since sex was found to be a significant predictor in the first overall tested model, and we also found differences in the correlation pattern of boys and girls (see Table 2). This decision was in accordance with several studies that found significant differences by sex in MC and HRF from early ages [5,28–32].

**Table 4:** Standard regression of MC components on HRF by gender and age groups.

**Boys**													
	**Height**		**BMI**		**Stability**		**Locomotor**		**Manipulative**			**Model**	
**Age**	**β**	**p**	**β**	**p**	**β**	**p**	**β**	**p**	**β**	**p**	**F**	**R**^**2**^_**adj**_	**p**
**7–8**	0.33	.000	-0.18	.034	0.16	.056	0.21	.035	0.36	.001	23.99	0.60	.000
**9–10**	0.15	.152	-0.12	.322	0.19	.874	0.49	.000	0.25	.023	12.10	0.44	.000
**11–12**	0.40	.000	-0.02	.872	0.50	.001	0.14	.315	-0.02	.898	20.89	0.61	.000
**13–14**	0.31	.004	0.00	.991	0.54	.000	0.02	.888	0.14	.249	17.15	0.60	.000
**Girls**													
	**Height**		**BMI**		**Stability**		**Locomotor**		**Manipulative**			**Model**	
**Age**	**β**	**p**	**β**	**p**	**β**	**p**	**β**	**p**	**β**	**p**	**F**	**R**^**2**^_**adj**_	**p**
**7–8**	0.04	.671	0.11	.290	0.19	.095	0.49	.000	0.17	.113	18.49	0.53	.000
**9–10**	0.13	.252	-0.10	.384	0.08	.572	0.50	.000	0.22	.079	10.63	0.42	.000
**11–12**	0.13	.324	0.14	.358	0.16	.253	0.65	.000	-0.11	.437	5.55	0.30	.000
**13–14**	0.17	.065	0.29	.008	0.46	.000	0.47	.001	0.09	.782	13.86	0.51	.000

Note: shadow cells represent significant coefficients (p < .05)

Both boys and girls displayed similar and substantial variance of the dependent variable (HRF) on the four age related models (girls: 30% to 53%; boys: 44% to 60%). When looking for the effect of the individual components of MC, we can see that the locomotor effect was particularly effective predicting HRF on the youngest boys (7–8 and 9–10 years) and for girls of all age groups. Manipulative control of objects was found to be predict HRF for boys in the first two age groups (7–8 and 9–10 years). Stability was a significant predictor for the two older age groups of boys (11–12 and 13–14 years) and for the oldest girls (13–14 years).

## Discussion

This study investigated the relationship between MC components and HRF in a large sample of 7 to 14 year-old children. In order to take into account developmental (age) and sex differences, four distinct age groups by sex were analyzed. The results showed positive moderate to high associations between MC and HRF regardless of age or sex. This relationship can be explained by the role that engagement in physical activity may have on the development and maintenance of both MC and HRF [16,18]. It is known that higher MC increases the participation in physical activities [33]. Since multiple aspects of neuromuscular development are integrated into both MC and HRF, these associations probably reciprocally influence each other [9]. Our results were consistent with previous research [16,18,34,35], but the correlations were generally higher, particularly in the older age groups [29,36,37]. Discrepancies in research findings about the relationship between MC and HRF can be attributed to the methods for measuring MC and HRF. In the present study, we used three different components of MC as defined by the theory of motor development [10], and objectively measured motor tasks with no age ceiling effect. The Pacer test and a hand dynamometer were used to assess HRF (cardiorespiratory fitness and strength). These tests objectively measure the intended component, so there was less room for cross-contamination on the nature of the assessment.

Locomotor MC had the most significant relationship to HRF in both boys and girls. This is probably due because cardiorespiratory fitness, one of the components of HRF, is heavily dependent on locomotor running skills for its development in childhood and adolescence. The correlations between the manipulative component of MC varied by sex, with boys demonstrating a higher correlation with HRF than girls. This may be explained by gender differences in sports participation in the Portuguese society where girls are usually less prone to participate in traditional team sports, while boys are encouraged to do so.

The present study also demonstrated that the relationship between MC and HRF was stable across time in both sexes. In fact, a tendency towards a clear strengthening or weakening of the correlation between MC and HRF over time was not detected, as opposed to studies that reported stronger correlations among young adults ages 18 to 25 years [38]; or studies that found a decrease in correlation values from childhood to adolescence [36,37]. Recently, Stodden and colleagues [17] presented findings in support of the developmental model with children 4 to 13 years old. However, the authors used only two manipulative tasks and one locomotor task versus a MC composite as used in this study. Stodden and colleagues (17) attributed the strengthening of the relationship between MC and HRF over time to the increased strength of the association between manipulative skills and HRF. However, in our separated sex analyses, no clear tendency was found. Instead, correlation values between MC components and HRF can be interpreted as stable with a tendency for locomotor and stability skills to have higher correlations than manipulative skills in all age groups for both sexes. To our knowledge, few studies analysed the relationship between the different MC components and HRF. Our results are consistent with previous research examining manipulative and balance components [16,36]. However, we did not find any studies that analysed the locomotor component independently.

When testing for the effect of MC on HRF with the entire sample, multiple regression analyses showed that the MC composite and MC components could predict a reasonable amount of HRF variance (74%), higher than the results (65% explained variance) obtained by Stodden and colleagues [17] in a similar study. This may be explained by the use of a more complete MC assessment in this study than the three motor skills used by the aforementioned authors (kick, throw, and jump).

Interestingly, when looking for the individual components of MC, locomotor skills presented the highest explained variance (see beta values in Table 3). This is contrary to what had been previously reported for a similar population [17], but similar to the results found in young adults aged 18–25 [38].

One important finding of the multiple regression analyses by age group was that sex differences were found in the independent predictor MC components. Manipulative and locomotor skills were the most consistent significant predictors for the two younger age groups of boys. For girls, the locomotor component of MC was the more relevant predictor for girls’ HRF. The stability component changed with age and sex, gaining influence with age to become the most influential predictor for the two older groups of boys (11–12 and 13–14 years) and the older group of girls (13–14 years). Therefore, our results did not support Barnett and colleagues' [24] suggestion that manipulative skills are the better indicator to explain associations between MC and HRF across childhood and into adolescence. Instead, they suggest a differentiated sex pattern in the development of the composite MC and its influence over HRF, probably influenced by the differentiated gender associated motor activities that girls and boys participate in daily.

Overall, the results of our study show that MC has an influential role in the development of HRF during childhood. Working with children to develop motor competence from an early age will trigger the achievement of better health related fitness, probably by the involvement of children in physical activities, sports and free play activities. The sex differences found between the MC components that were found to have a significant relationship to HRF, suggests that boys and girls do not share the same kind of motor activities. For girls, locomotor motor competence (quality of running, jumping, etc.) may be important to trigger their involvement in physical activities. For boys, both locomotor and manipulative motor competence showed to be relevant for the initial involvement in motor activities, probably because ball games provide both types of benefits. It may also be that the quality of the stability component (dynamic body balance) assures more success on motor activities.

Since childhood is a critical period for the acquisition of MC and HRF, it should be fundamental to promote both MC and HRF to benefit a healthy development of children.

The present study extends the previous work developed by Stodden and colleagues [17]. To our knowledge, this is the first study to analyze the relationship between MC and a composite HRF index across a large sample of male and female children and adolescents, using a validated instrument [12] that includes the three theoretical components proposed by Gallahue and colleagues [10]. However, the current study presents some limitations. The cross-sectional design of the present study does not provide definitive causal evidence with respect to the relationship between MC and other variables. To gain more insight into the direction of this relationship, longitudinal and intervention studies should be conducted. The absence of physical activity, sedentary time, and maturational information (*e*.*g*., sexual maturity, skeletal maturity) are limitations of this study. Physical activity and sedentary time, if measured, could have been included as covariates in the analysis, given the more than likely association with both HRF and MC. Biological maturation influences all variables especially during the transition from childhood to adolescence; therefore, this variable should have been taken into consideration. Nevertheless, the inclusion of height and BMI as independent variables allowed for a possible control of maturational effects on HRF. Despite the positive associations between handgrip task with both maximal upper and lower body strength [39], other variables could have been included for this purpose, such as the vertical jump to assess leg strength.

## Conclusions

To our knowledge this study is the first that analyzed the relationship between motor competence composite and its three theoretical components with health-related fitness in a large sample of children using a valid quantitative instrument. Our results support the idea that MC and HRF are closely related in both boys and girls from 7 to 14 years and that this association is stable across ages, contrary to previous findings [17]. All MC categories presented similar trends to the MC composite, with locomotor and stability skills being more frequently and strongly associated with the HRF index.

This study also intended to look for the effects of MC and its individual components (independent variables) for explaining HRF (dependent variable). All MC components proved to be significant predictors of HRF, with the locomotor component being the strongest predictor. Furthermore, separate analyses for boys and girls in each age group indicated different significant predictors according to age. For girls, the locomotor component of MC clearly had the strongest effect on HRF across all ages; while locomotor and manipulative components showed bigger effects in younger boys and stability in the older ones. Stability skills seem to become important predictors of HRF with growing age for both sexes. Studies using longitudinal information are needed to further disentangle this effect of MC components across developmental ages.

School-based interventions should consider the development of MC and its components as a key strategy to promote a healthy development across the lifespan.
